# Bidirectional causal control in the dynamics of handstand balance

**DOI:** 10.1038/s41598-020-79730-z

**Published:** 2021-01-11

**Authors:** Hannah E. Wyatt, Domenico Vicinanza, Karl M. Newell, Gareth Irwin, Genevieve K. R. Williams

**Affiliations:** 1 Sports Performance Research Institute New Zealand, Auckland University of Technology, Auckland, New Zealand; 2 School of Computing and Information Science, Anglia Ruskin University, Cambridge, UK; 3 Department of Kinesiology, University of Georgia, Athens, USA; 4 Cardiff School of Sport and Health Sciences, Cardiff Metropolitan University, Cardiff, UK; 5 School of Sport and Health Sciences, University of Exeter, Exeter, UK

**Keywords:** Nonlinear dynamics, Biological physics

## Abstract

The aim of this study was to identify motor control solutions associated with the ability to maintain handstand balance. Using a novel approach, we investigated the dynamical interactions between centre of pressure (CoP) and centre of mass (CoM) motion. A gymnastics cohort was divided into a ‘less skilled’ group, who held handstands for 4–6 s, and a ‘more skilled’ group, who held handstands in excess of 10 s. CoP–CoM causality was investigated in anterior–posterior (AP) and medio-lateral (ML) directions, in addition to time–space, time–frequency and Hurst Exponent (*H*) analyses. Lower AP CoP to CoM causal drive and lower *H* values (> 0.6) indicated the more skilled gymnasts were less reliant on CoP mechanics to drive CoM motion. More skilled performance demonstrated greater adaptability through use of reactive, as opposed to anticipatory, control strategies. Skilled performers additionally exploited mechanical advantages in ML (e.g. a wider base of support), compared to the less skilled athletes. A multiple regression analysis revealed *H* and frequency domain measures to be better predictors of handstand balance duration than time–space domain measures. The study findings highlight the advantage of an adaptable motor control system with a directional profile, and provide new insight into the clear, measurable footprint of CoP on the dynamics of CoM.

## Introduction

Based on current understanding, the complexity and redundancy of human movement outdoes our best abilities to model its mechanics. Commonly used methods employed to understand motor control, including correlations, phase relations and coherence, have made limited advances in the attempt to capture the ubiquitous nonlinearity of systems in nature. Determination of the extent to which one system drives the behaviour of another through causality analysis may elegantly uncover the mechanical interactions which underpin motor control during balance.

The causality approach provides a framework that can be generalised across nonlinear systems in ecology. Identifying causal relationships, characterising their nature and quantifying their strength extends beyond human balance, it is in fact a general problem of many complex dynamical systems. For example, methods based on the idea of nonlinear attractor reconstruction to observed time series have been successfully applied to model simulations, temperature and greenhouse gas reconstructions from the Vostok ice core and long-term ecological time series collected in the Southern California Bight^1^. Here, in the first use of causality in human nonlinear dynamics systems, we examine coupled centre of pressure (CoP)—centre of mass (CoM) motion to translate these ideas to understand causal relationships as part of human motor control.

Our vehicle for a human motor control problem is the handstand balance. A handstand is defined as the act of holding the body in an inverted vertical stance with the hands in contact with the support surface. Although considered a fundamental skill in sports such as gymnastics, the organisation of inverted posture requires complex multivariate coordination^2^ and reflects the mastery of many redundant degrees of freedom^3^. The performance of a handstand requires simulation of the basic human action of upright posture and creates an interesting landscape for the study of motor control. The common goal of preserving balance with respect to gravity elicits different challenges when balancing on two arms instead of two legs, including structural capabilities, a higher CoM and smaller surface area^4–7^. A reduction of the base of support leads to greater postural variability than upright stance, hence the inverted posture imposes greater stresses on the system and more revealing control strategies.

Transitioning from upright standing posture to inverted stance, or ‘handstand balance’, relies initially on large changes in body positioning and once inverted, on subtle changes of hand pressure and limb actions to control posture during the stabilising period. The performer is required to transition through a period of instability to reach a balanced posture. Interplay between the numerous neurophysiological and biomechanical control processes are anticipated to be vital to the duration of handstand balance and have the potential to differentiate more and less successful handstand performance. Previous analysis of handstand biomechanics has investigated control strategies including the coordination of joints and segments^4–6,8^, the role of vision^9^ and perturbed balance^10^. Particular emphasis has been placed on anterior–posterior (AP), sagittal plane mechanics^5,8,10^; consequently we have little understanding of gymnasts’ medio-lateral (ML) control mechanisms. Biomechanical analyses used to further understand handstand balance control have been centred on individual and coordinated structures, primarily the hips, shoulders and wrists; however, the role of macroscopic variables, such as the CoM and CoP, in the maintenance of handstand balance is largely unknown, despite being a focus of upright stance biomechanics.

Unpacking the characteristics of CoM and CoP motion during upright stance has provided valuable insight into balance strategies and their underlying motor control mechanisms^11–15^. Regulation of CoM positioning has been identified as being crucial to the maintenance of balance^16^, while CoP magnitudes have been directly associated with postural instability in cohorts with balance disorders^17,18^. Additional insight into mechanisms of postural control have been achieved from the analysis of signal frequencies. For example, previous research by Schinkel-Ivy et al.^19^ has outlined an insightful approach to interpret ‘anticipatory’ postural adjustments as slower at < 0.4 Hz and ‘reactive’ adjustments as faster at > 0.4 Hz during transient instability. This frequency thresholds as well as the use of the terminology “reactive” as opposed to “compensatory” control is employed to distinguish these mechanisms with reference to our handstand data.

Biological systems are characterised by complex nonlinear dynamics^20^. Capturing how the system (or signals from the system) evolve in time reveals important features of postural stability which are not exposed through linear analyses in the time or frequency domains, as exemplified in standing posture by Blaszczyk and Klonowski^21^. Further understanding of signal complexities and finer-grained dynamics of mechanisms involved in the control of posture have been demonstrated in upright standing through Hurst exponent (*H*) nonlinear dynamics analysis of central variables such as CoP^21–23^. *H* outputs demonstrate the characteristics of signal evolution over time through the presence of anti-persistence or long-range correlations. In addition, relative signal magnitudes provide a window into the motor control of the system, where lower fractal dimensions, thus long range correlations, show loss of variability in system; such dynamical characteristics have been associated with ageing^23^. It is hypothesised that less variability might be present in the CoP signal of less skilled handstand performance due to the use of anticipatory, rather than reactive control.

There are both mechanical and motor control reasons to move beyond independent CoM and CoP analyses to the explore the relationship between CoM and CoP motions. For example, Newell and colleagues highlighted CoM–CoP relative phase as a promising candidate for a collective variable in upright standing posture^24,25^. The work undertaken in upright posture provides evidence that the relationship between CoM and CoP motion may be associated with important aspects of control in handstand balance. CoM and CoP are a coupled system where CoP captures forces applied to the ground, which along with segmental motions, are used to maintain the CoM within the base of support. Reciprocally, CoM motions project onto CoP. However, a limitation to capturing the phase relationships between CoM–CoP is the incomplete transient aspects and the cause of the relationship. Using a novel approach, we examine the causal links between CoM and CoP in order to capture what is closer to the actual ‘motor control’ of the situation. That is, to identify to what extent the behaviour of the CoP drives the behaviour of the CoM, or vice versa, and enable us to conclude the strength of their mechanical and dynamical relationship.

**Figure 1:**
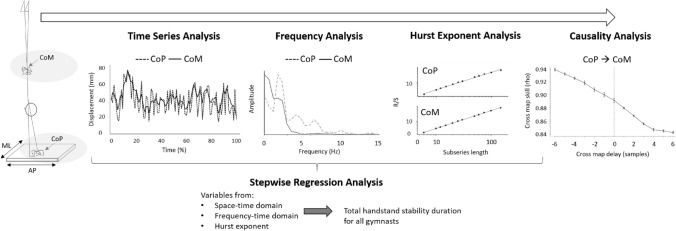
An overview image of the data analysis process to investigate CoP and CoM motion during the stabilising phase of a handstand for more skilled and less skilled gymnasts, in addition to a balance phase for the more skilled gymnasts. A representative trial of CoP and CoM is provided to illustrate each form of analysis.

Therefore, the aim of this work was to identify motor control solutions associated with the ability to maintain handstand balance. To address the study aim, CoM and CoP motion during handstand performances were analysed for two groups: a more skilled group, who were able to hold handstands in excess of 10 s, and a less skilled group, who held handstands for 4–6 s. A balance phase (6–9 s) of the handstand was also investigated for the more skilled gymnasts’. Causality analysis was performed to identify the extent to which CoP drives or is driven by CoM motion. More traditional analysis in the time domain, AP and ML CoM and CoP displacements and velocities, frequency domain, AP and ML CoM and CoP power < and > 0.4 Hz, and power spectrum centroid were examined in addition to nonlinear dynamic *H* (Fig. 1). A multiple stepwise regression was undertaken to establish the model with greatest contribution to handstand balance duration.

## Results

We defined the start of the handstand when the minimum sagittal distance between the left and right feet was reached. From the start of the handstand, CoM and CoP data for the initial 3 s, referred to as the stabilising phase, were analysed for each trial and participant. Balance phase data were additionally analysed using a 3 s period during the more skilled gymnasts’ handstand (6–9 s).

### Time domain

**Figure 2:**
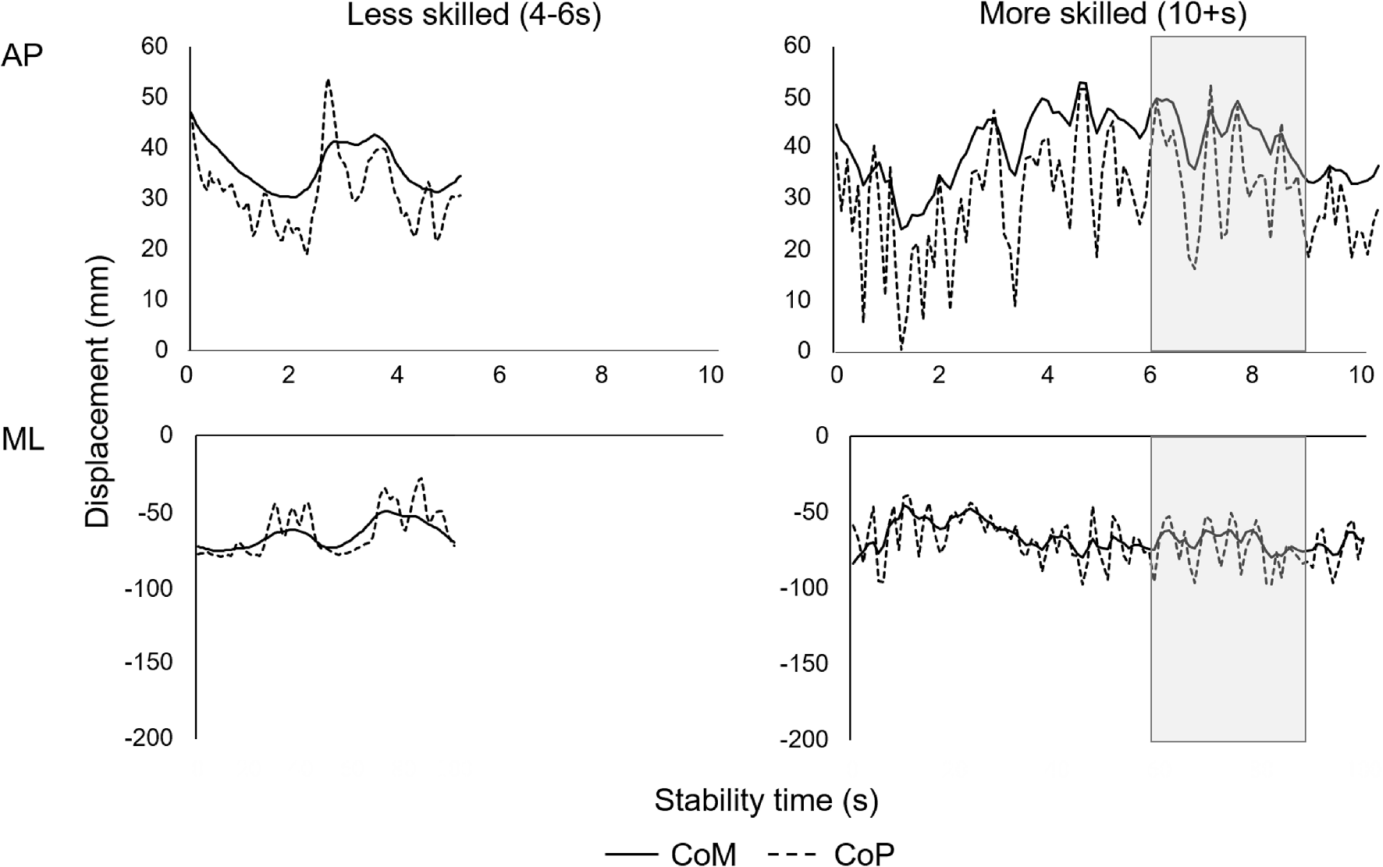
CoM and CoP AP and ML displacement for example trials from a less skilled and a more skilled gymnast. Shaded periods indicate the data phases used for more skilled balance phase analysis (6-9 s).

Handstand duration was significantly greater for the more skilled than less skilled gymnasts (*P* < 0.001). Figure 2 includes continuous positional data of one trial for a single gymnast in each of the two groups to provide insight into CoM and CoP space–time interactions.

Discrete CoM and CoP displacement and velocity outputs were documented for the more and less skilled stabilising phase and the more skilled balance phase. Both groups (less and more skilled) had greater AP and ML displacements of CoM and CoP during the stabilising phase than the more skilled gymnasts’ balance phase.

Mean and maximum instantaneous velocity were greater for the stabilising phase than the more skilled balance phase in each direction (AP and ML), other than for the less skilled gymnasts’ maximum CoM instantaneous velocity in AP (Table 1). The more skilled gymnasts had significantly greater mean AP CoP velocity during the stability phase than the less skilled gymnasts (15.6 mm s^−1^ difference, *P* < 0.05).

**Table 1:** Time domain displacement range and velocity outputs for CoM and CoP mean ± s.d. for each group.

	CoM			CoP		
	Stabilising phase	Stabilising phase	Balance phase	Stabilising phase	Stabilising phase	Balance phase
	Less skilled	More skilled	More skilled	Less skilled	More skilled	More skilled
AP displacement range (mm)	**25.7 ± 8.8**^**ac**^	24.6 ± 7.7	**18.4 ± 7.1**^**ac**^	**54.1 ± 10.1 **^**ac**^	**57.0 ± 12.2**^**bc**^	**44.8 ± 9.2**^**ac,bc**^
ML displacement range (mm)	**24.1 ± 13.4**^**ac**^	**18.3 ± 7.8**^**bc**^	**12.9 ± 7.3**^**ac,bc**^	47.4 ± 23.2	43.8 ± 20.0	34.2 ± 13.8
AP mean velocity (mm·s^-1^)	18.4 ± 3.4	**20.6 ± 5.0**^**bc**^	**15.8 ± 4.3**^**bc**^	**88.8 ± 13.7**^**ab**^	**104.4 ± 16.1**^**ab,bc**^	**81.5 ± 13.5**^**bc**^
ML mean velocity (mm·s^-1^)	**15.0 ± 4.6**^**ac**^	**12.4 ± 2.2**^**bc**^	**9.5 ± 2.8**^**ac,bc**^	59.9 ± 11.0	**72.1 ± 12.0**^**bc**^	**48.7 ± 12.2**^**bc**^
Maximal AP instantaneous velocity (mm·s^-1^)	67.8 ± 14.4	95.8 ± 66.9	70.7 ± 53.5	**390.9 ± 70.1**^**ac**^	**395.0 ± 71.0**^**bc**^	**319.3 ± 62.2**^**ac,bc**^
Maximal ML instantaneous velocity (mm·s^-1^)	42.6 ± 12.5	44.6 ± 21.7	32.3 ± 13.6	237.8 ± 81.9	**323.2 ± 101.9**^**bc**^	**180.7 ± 59.3**^**bc**^

Bold values indicates a significant difference between two or more conditions (*P* < 0.05).

^ab^*P* < 0.05 between less and more skilled stabilising phase (independent t-test).

^ac^*P* < 0.05 between less skilled stabilising phase and more skilled balance phase (independent t-test).

^bc^*P* < 0.05 between more skilled stabilising phase and more skilled balance phase (paired t-test).

### Frequency domain

A banding approach using a 0.4 Hz threshold was selected due to its identification as a biomechanically important frequency band which provides insight into anticipatory and reactive control strategies within standing posture^19^. The 0.4 Hz frequency threshold was used to investigate signal power across frequencies (Table 2). More skilled gymnasts had greater ML CoP power at frequencies above 0.4 Hz than less skilled gymnasts (22.31% difference, *P* = 0.007, effect size (ES) = 1.07). Power was greater above 0.4 Hz during the more skilled balance phase compared to the less skilled gymnasts’ stabilising phase (15.61%, *P* = 0.019, ES = 0.91). Power spectrum centroid values for CoP did not differ between more and less skilled gymnasts and the more skilled gymnasts’ balance phase (*P* > 0.05).

**Table 2:** Frequency domain power < and > 0.4 Hz and power spectrum centroid for CoM and CoP mean ± S.D. for each group.

	CoM			CoP		
	Stabilising phase	Stabilising phase	Balance phase	Stabilising phase	Stabilising phase	Balance phase
	Less skilled	More skilled	More skilled	Less skilled	More skilled	More skilled
AP power < 0.4 Hz (%)	99.71 ± 0.64	97.90 ± 7.33	99.55 ± 1.39	98.05 ± 3.11	96.04 ± 9.48	95.56 ± 15.01
ML power < 0.4 Hz (%)	98.67 ± 2.90	94.54 ± 9.15	96.81 ± 3.81	**94.82 ± 5.61**^**ab,ac**^	**72.19 ± 29.29**^**ab**^	**78.99 ± 24.01**^**ac**^
AP power > 0.4 Hz (%)	0.29 ± 0.64	2.10 ± 7.32	0.45 ± 1.39	1.94 ± 3.09	3.92 ± 9.38	4.37 ± 14.77
ML power > 0.4 Hz (%)	1.33 ± 2.90	5.46 ± 9.15	3.18 ± 3.80	**5.12 ± 5.54**^**ab,ac**^	**27.43 ± 28.92**^**ab**^	**20.73 ± 23.68**^**ac**^
AP power spectrum centroid	–	–	–	1.36 ± 0.10	1.36 ± 0.09	1.37 ± 0.09
ML power spectrum centroid	–	–	–	1.28 ± 0.09	1.33 ± 0.14	1.34 ± 0.12

Bold values indicates a significant difference between two or more conditions (*P* < 0.05).

^ab^*P* < 0.05 between less and more skilled stabilising phase (independent t-test).

^ac^*P* < 0.05 between less skilled stabilising phase and more skilled balance phase (independent t-test).

### Nonlinear dynamics

*H* outputs revealed the tendency for long range correlations (> 0.6) that were on a shorter scale within the AP CoM signals and the ML CoP signals, indicating less predictability, for the more compared to the less skilled group (0.05 and 0.07, respectively; Table 3). Significantly longer long-range correlations, indicating a more predictable signal, were identified for the less skilled group compared with the more skilled balance phase for ML CoM and AP CoP (0.06 and 0.13 differences, respectively).

**Table 3:** Hurst exponent (*H*) outputs for CoM and CoP mean ± S.D. for each group.

	CoM			CoP		
	Stabilising phase	Stabilising phase	Balance phase	Stabilising phase	Stabilising phase	Balance phase
	Less skilled	More skilled	More skilled	Less skilled	More skilled	More skilled
AP *H*	**0.92 ± 0.04**^**ab,ac**^	**0.87 ± 0.05**^**ab**^	**0.88 ± 0.06**^**ac**^	**0.74 ± 0.06**^**ac**^	**0.73 ± 0.06**^**bc**^	**0.61 ± 0.03**^**ac,bc**^
ML *H*	**0.94 ± 0.04**^**ac**^	0.91 ± 0.05	**0.88 ± 0.06**^**ac**^	**0.77 ± 0.05**^**ab**^	**0.70 ± 0.07**^**ab**^	0.74 ± 0.03

Bold values indicates a significant difference between two or more conditions (*P* < 0.05).

^ab^*P* < 0.05 between less skilled and more skilled stabilising phase (independent t-test).

^ac^*P* < 0.05 between less skilled stabilising phase and more skilled balance phase (independent t-test).

### Causality analysis

CoP was found to drive CoM consistently across all conditions studied (Table 4). The more skilled gymnasts had a significantly weaker CoP–CoM causal link in AP compared to the less skilled gymnasts (0.02 difference, *P* < 0.05), indicating that during the stabilising phase, the more skilled gymnasts relied on CoP to control CoM motion to a lesser extent than the less skilled gymnasts.

**Table 4:** CoP to CoM mean ± S.D. causality for ML and AP.

	CoP → CoM		
	Stabilising phase	Stabilising phase	Balance phase
	Less skilled	More skilled	More skilled
AP Causality	**0.98 ± 0.02**^**ab**^	**0.96 ± 0.03**^**ab**^	0.96 ± 0.05
ML Causality	0.97 ± 0.05	0.98 ± 0.03	0.94 ± 0.09

Bold values indicates a significant difference between two or more conditions (*P* < 0.05).

^ab^*P* < 0.05 between less and more skilled stabilising phase (independent t-test).

### Stability time regression outputs

Of the linear and nonlinear dynamic variables that differed significantly between the less and more skilled gymnasts, ML CoP *H* accounted for the greatest proportion of variance in handstand stability duration, i.e. the length of time the gymnasts were able to maintain an inverted stance position (R^2^ = 0.463). ML CoP Power < 0.4, ML CoP Power > 0.4, AP CoM *H* and ML CoP velocity explained 27%, 27%, 21% and 20% of stability time, respectively.

When considered by way of a stepwise multiple regression, ML CoP *H* and ML CoP Power < 0.4 collectively explained 63% of handstand stability time (R^2^ = 0.629, *P* < 0.001).

## Discussion

The aim of this study was to identify motor control solutions that are associated with the ability to maintain handstand balance. That is, to investigate the dynamical interactions between CoM and CoP motion. Causal drive of CoP to CoM (i.e. the extent to which CoM motion was driven by CoP) was found to be directionally dependent (AP and ML). We found the more skilled gymnasts to be characterised by lower causal drive from CoP to CoM in the AP direction, with shorter-range correlations (or shorter memory) of CoM and CoP signals, suggesting that segmental actions, rather than CoP alone, were key to maintaining balance. In the ML, CoP–CoM drive was more common in the skilled and stable condition (balance phase), revealing it is favourable for the simpler mechanical relationship to be exploited in ML direction.

The application of causality analysis here provides a framework that can be used in other situations to understand ecology and motor control. Specifically, we suggest that this method transcends information gained from pure mechanics or simply unpacking the dynamics of individual signals. It is particularly potent in situations where we know fundamental mechanical links do exist, but we also need to acknowledge subtle, redundant interactions ubiquitous of biological systems. The CoP–CoM relation in balance is an example, as well as other motor control phenomenon such as limb interaction in gait. Understanding the ways in which nonlinear causal networks underpin the search for explanations or interpretations of complexities outside of these relationships are likely inherently related to skill level or health status in motor control. This can expand the application of dynamical system causality in studying other phenomena like the loss of balance in ageing, fatigue or disease.

Providing unique insight into motor control, causal links between the coupled motion of the CoM and CoP revealed that in the AP direction, the more skilled group had a significantly lower causal drive from CoP to CoM, compared to those less skilled (Table 4). It is suggested that there is greater redundancy in the CoP–CoM couple for the more skilled trials, where CoM motion is controlled by compensatory actions at the segmental level that do not project from the CoP alone. This idea is supported by previous work on handstand balance which identifies wrist and elbow motion as strategies to minimise motion of CoM^10^. Such strategies have greater subtlety than those of the hips which are influential in less successful balances^5^ and may lessen the causal influence of the CoP to the CoM motion based on a compensatory strategy within limbs.

In examining the structure of the CoM and CoP signals, long range correlations dominated the CoM and CoP signals (*H* > 0.6, Table 3). A lower *H* output for the more skilled group’s CoM in AP is indicative of shorter-term memory in the CoM motion. On the contrary, CoM *H* values close to 1 (0.92 for AP and 0.94 for ML) for less skilled gymnasts suggests that the underpinning mechanical system is driving the evolution of the CoM so strongly that there is very little room for reactive corrections. Overall, the findings suggest that the more skilled group had ‘freed’ the association between the CoM and CoP in the AP direction and had shorter memory in CoM dynamics. Greater skilled performance appears to be characterised by motor control subtleties that define the relations of CoM and CoP in a more independent and complex way in the AP direction. The ability to manipulate the two macroscopic variables could be more advantageous based on greater redundancy in the system to counteract periods of potential instability.

Motor control characteristics differ in the AP and ML direction, based on the degrees of freedom that operate in the sagittal and frontal plane^4^. ML CoP to CoM drive was more common in the skilled and stable conditions (balance phase), suggesting ML CoP drive may be an important mechanism for high level, long lasting handstand balance. Explained by the limited segmental actions that take place in the frontal plane, as well as the larger base of support that creates the opportunity for larger rotational torques as a result of the hand contact with the ground, a simpler less redundant mechanical mechanism is used where CoP more directly causes CoM motion in ML. Future work might explore the effect of hand position on CoP–CoM driving dynamics, limiting base of support to examine changes in control strategy and may seek to better understand the complexities of the interplay between different systems (e.g. muscular) and postural control during the handstand balance.

In addition to a causal drive to CoM, in ML the more skilled group had higher power at higher frequencies in CoP oscillations compared to the less skilled group. Upright posture literature has provided insight into the type of control mechanisms used through analyses of associated power across frequency ranges. Anticipatory control mechanisms, typically adopted for the use of an exploratory approach using intentional input that leads to increased errors in state estimation, have been associated with increased power at lower frequencies^19^, as was found for the less skilled group for ML CoP. Reactive control mechanisms, whereby an individual makes corrections in response to transient instability, has been associated with increased power at higher frequencies, as was found for the more skilled group for ML CoP. The more skilled performers additionally had more complex CoP signals, with lower *H* (less predictability) in ML compared to the less skilled group. In summary, the more skilled gymnasts’ ML CoP signal was faster oscillating and less predictable than that of the less skilled gymnasts.

The sensitivity of nonlinear and frequency measures to handstand balance performance have been evidenced to be greater than that of space-time for skill level. Based on the regression, *H* distinguished more and less skilled groups to the greatest extent. Contemporary nonlinear dynamics analyses, including *H* and causality, have provided greater depth of understanding for the characteristics of CoM and CoP during handstand balance and therefore inform valuable insight into motor control solutions associated with the ability to maintain handstand balance. Potentially important features of control during a stabilising activity may be omitted when analyses are limited to commonly used space-time measures^13,23^.

Biomechanically important frequency bands which provide insight into proactive and reactive control strategies (0.4 Hz cut-off^19^) have been determined within quiet stance, however this is yet to be studied in inverted posture. The frequency banding approach was taken in the current study in alignment with standing literature, however, further study of frequency bands which are important for handstand control would make an important contribution to understanding successful handstand balance performance. The current data set was limited by the number of data points, due to sampling rate (100 Hz) and capture time (3 s). The scope of the analyses with the identified data set limited wavelet transform analysis to below 1.1 Hz.

## Conclusion

Physical systems with time-evolving internal couplings (such as CoM and CoP) are commonplace in nature. While the analytical characterisation of these systems (i.e. their governing equations) is generally not possible to determine due to insufficient understanding of their internal mechanisms, the application of causality based on non-linear attractor reconstruction provides an ideal framework to detect causal relationships between their linked components^26^. The idea to identify and characterise the underlying physical mechanisms governing the relationship between two subsystems, CoM and CoP, can be used in other situations within ecology and motor control. The specific analysis approach is particularly suitable for situations where fundamental mechanical links exist, but there is a need to acknowledge subtle, complex and redundant interactions ubiquitous to biological systems. Through understanding of nonlinear causal networks, we can drive the search for explanations or interpretations of complexities outside of these relationships which might be inherently related to skill level or health status in motor control.

In our handstand example, greater skilled performance was characterised by subtleties that define the relations of CoM and CoP in a more independent and complex way in the AP direction. The ability to manipulate the two macroscopic variables could be more advantageous based on greater redundancy in the system, counteracting periods of potential instability. In the ML direction, gymnasts can rely on mechanical support from a wide base of support when CoP drives CoM motion. Skilled performance is therefore suggested to be reliant on exploiting the best use of mechanical and dynamical associations through a more constrained ML and a more ‘free’ AP motor control solution. Understanding causal links between CoM and CoP provides a new approach to investigating postural stability which captures both the mechanics and the dynamics of the system.

## Methods

### Participants

Competitive female artistic gymnasts who trained regularly at a national level were recruited for the study (mean age = 10 ± 1 years, height = 1.37 ± 0.09 m, mass = 31.5 ± 2.9 kg, training duration = 5 ± 2 years, training frequency = 20 ± 5 h/week). Of the ten participants that were recruited, five gymnasts were able to maintain handstand position for a maximum duration of 4–6 s (less skilled gymnasts) and five maintained a handstand position in excess of 10 s (more skilled gymnasts). No participant demographic characteristics differed significantly between groups (*P* > 0.05).

As previously detailed^27^, the recruitment inclusion criteria required gymnasts to be injury free, training regularly, and to have taken part in a minimum of one competition (club to international level) in the preceding 12-month period. A 9–15 years age criteria was established at recruitment, in accordance with the 2015 National British Gymnastics competition lower age boundary and upper junior age boundary, respectively. Ethical approval for the collections was obtained from Cardiff Metropolitan University’s Research Ethics Committee. All participants and their parents (or guardians) provided written informed consent and all methods were performed in accordance with the relevant ethics guidelines and regulations.

### Experimental setup

Kinematic data were recorded using four CODA motion Cx1 units (Charnwood Dynamics Ltd., Leicestershire, UK) sampling at 100 Hz, synchronised with ground reaction force data from a Kistler force plate (9287BA, Kistler, Switzerland) operating at 1000 Hz. Forty-eight active markers were fitted bilaterally to each gymnast on the lower extremities (n = 12), pelvis (n = 6), lumbar spine (n = 7), torso (5) and upper extremities (n = 18). Participants wore shorts and a cropped top to allow for maximal marker placement directly onto the skin.

### Protocol

Each gymnast performed 20 handstand trials as they would within a typical training session. Handstands were performed onto a Mondo-covered force plate and inverted stance was maintained for as long as possible with a maximum duration of 25 s. Trials were rejected if full force plate contact with both hands was not sustained throughout the inverted stance. Participants were instructed to take as much recovery time as they needed between trials to minimise any effects of fatigue.

### Data processing and analysis

CoP data were calculated in Visual 3D software (v6, C-motion, Inc., Rockville, MD) for the inverted stance durations of three trials for each gymnast. Computed from the force platform analog signal and resolved in the laboratory coordinate system, CoP was representative of the average position of all external forces acting on the palmer surface of the hand. Whole-body CoM was calculated as a weighted average of 15 segments (feet, shanks, thighs, pelvis, lumbar spine, torso, hands, forearms and upper arms), using individual Visual 3D models, in accordance with previous work^27^.

Inverted stance initiated when the minimum sagittal distance between the left and right feet was reached. From this point, the initial 3 s of handstand balance was analysed for each trial and all participants (more and less skilled gymnasts). For trials which were maintained in excess of 10 s (more skilled gymnasts), a period of handstand maintenance between 6 and 9 s was analysed (referred to as the more skilled balance phase). Continuous AP and ML CoM and CoP data were exported at 100 Hz for each trial in each condition (more and less skilled stabilising phase and more skilled balance phase).

Within the space–time domain, displacement range, mean velocity and maximum instantaneous velocity were calculated for AP and ML motion. To explore the frequency-time characteristics of the CoM and CoP signals, power spectral density (PSD) in 1.5 Hz bands of the spectrum were analysed within R-Studio (RStudio, Inc; https://www.rstudio.com, seewave library). The estimation of the PSD was based on Bartlett’s method of averaged periodograms, using non-overlapping Hanning windows of 512 points (approximated a 0.1953 Hz frequency bin for each power spectral estimate). Using PSD, the percentage of power above and below a 0.4 Hz threshold was calculated for each trial and variable.

*H* estimation was used to explore structural characteristics of the CoM and CoP time series via part of the DFA function from the fractal R library, (https://rdrr.io/cran/fractal/man/DFA.html), with minimum block size to partition the data set to 6 points. The fractal analysis provides a measure for long-term memory (and predictability)^28^, requiring very few assumptions about the underlying system. Values of *H* range between 0 and 1 where *H* = 0.5 indicates a random series. Values between 0 and 0.5 indicate a mean-reverting, anti-persistent time series (i.e. an increase in the time series is more likely followed by a decrease, and vice versa) for which the strength is greater as *H* approaches 0. Values between 0.5 and 1 show long-range correlation, with a trend reinforcing behaviour (a growth is likely to be followed by another growth) that increases as *H* approaches 1. In general, a time series with a large *H* are more predictable than those with *H* close to 0.5.

The existence of causal links, together with their strengths and direction, was determined using convergent cross mapping (CCM), a method introduced by Sugihara^29^ and based on Takens’ Theorem^30^. According to CCM if a variable x does influence another variable y, then the historical values of x can be recovered from variable y alone. In practical terms, a time delay embedding is constructed from y, and the ability to estimate the values of x from this embedding quantifies how much information about x has been encoded into y (i.e. how much of the dynamics footprint of x has been transferred to y). In this way we can quantify the causal effect of x on y is through how well y “cross maps” x.

In this paper we used an extended version of the original CCM algorithm, published in 2015 by Ye et al.^1^ to detect causal links between CoM and CoP dynamics. This extension makes it possible to uniquely determine whether a driving variable acts with some time delay on a response variable. This allowed us to exclude false positives when the apparent synchrony is actually caused by a strong unidirectional external force that drives both x and y variables and, in our case, clearly identify if and under which condition CoP drives or is driven by CoM. This will also exclude false positives due to pure mechanical synchronisation, where the CoM and CoP would be just rigidly linked. A footprint of one variable on the dynamics of the other, demonstrating clear causal drive, was indicated by a negative cross map delay. The mean group magnitude at the point of maximum delay (cross map skill) subsequently informed the causality data outputs (Table 4). We investigated the CCM in both directions (CoP to CoM and CoM to CoP). Our results have been computed using the ccm_udf implementation of the extended CCM algorithm^31^. The function automatically calculates the optimal Takens parameters for each CoM and CoP time series; our values ranged from 3 to 6 for the embedding dimension and from 9 to 12 for the time delays. False positives were not included in further statistical analysis. Monte Carlo methods were used to assess the statistical significance against noise. Specifically, we used a large ensemble of surrogate data set pairs (1000) with the same AR1 coefficients as the input datasets.

### Statistical analysis

Using SPSS software (IBM SPSS Statistics 25, SPSS Inc., Chicago, IL), the statistical differences between less skilled stabilising, more skilled stabilising and more skilled balance phases were analysed for handstand duration, each of the space–time and frequency-time domain variables, *H* and causality outputs. Statistical analyses between more and less skilled stabilising phase, and less skilled stabilising phase and more skilled balance phase were investigated using independent two-tailed t-tests; analysis between more skilled stabilising phase and more skilled balance phase were undertaken using paired two-tailed t-tests. Cohen’s d effect sizes were calculated for each statistical comparison. A stepwise multiple regression was run in SPSS with handstand duration as the dependent variable and all time, frequency and *H* variables which differed significantly between more and less skilled gymnast data input as independent variables. A criterion alpha of 0.05 was set a priori for all statistical tests.

## Data Availability

The datasets generated and/or analysed during the current study are available from the corresponding author upon reasonable request.
